# Counteractive Effects of Choline Geranate (CAGE) ILs and Ethanol on Insulin’s Stability—A Leap Forward towards Oral Insulin Formulation

**DOI:** 10.3390/molecules27155031

**Published:** 2022-08-08

**Authors:** Kandhan Palanisamy, Muthuramalingam Prakash

**Affiliations:** Department of Chemistry, Faculty of Engineering and Technology, SRM Institute of Science and Technology, Kattankulathur 603203, Tamil Nadu, India

**Keywords:** insulin dimer dissociation, CAGE IL, ethanol, water, molecular dynamics, well-tempered metadynamics, free energy landscape

## Abstract

Choline geranate (CAGE) ionic liquids (ILs) stabilize insulin, thereby aiding its oral delivery, whereas ethanol (EtOH) affects its stability by disrupting the hydrophobic interactions. In this study, cognizance of the stabilization mechanism of insulin dimer in the presence of both CAGE ILs and EtOH mixtures is achieved through biased and unbiased molecular dynamics (MD) simulations. Here, two order parameters are employed to study the insulin dimer dissociation using well-tempered metadynamics (WT-MetaD). The stability of insulin is found to be strongly maintained until a 0.20 mole fraction of EtOH. Besides, higher concentrations of EtOH marginally affect the insulin stability. Moreover, geranate anions form a higher number of H-bonding interactions with water molecules, which aids insulin stabilization. Conversely, the addition of EtOH minimizes the water-mediated H-bonding interactions of geranate. Additionally, geranate traps the EtOH molecules, thereby preventing the interactions between insulin and EtOH. Furthermore, the free energy landscape (FEL) reveals the absence of dimer dissociation along with noticeable deviations in the distances R and the number of contacts Q. The dimerization free energy of insulin was calculated to be −16.1 kcal/mol at a 0.20 mole fraction of EtOH. Moreover, increments in mole fractions of EtOH effectuate a decrease in the insulin stability. Thus, the present study represents CAGE ILs as efficient insulin dimer stabilizes at low concentrations of EtOH.

## 1. Introduction

In the realm of protein association and dissociation lies the center of diverse biochemical processes. Protein oligomerization is beneficial for some cases (e.g., insulin hexamer and hemoglobin), while some of the proteins form fibrils, which leads to neurodegenerative diseases [1,2]. Hence, it is integral to understanding the mechanisms of protein–protein association and dissociation, which occur simultaneously. These profound changes in the oligomerization of insulin and their implications in the biological processes need to be explored [3,4,5]. Insulin, a hormone peptide secreted from pancreatic β cells, is responsible for regulating the glucose levels in the blood as well as the normal metabolism of an organism [6]. Additionally, the incongruous secretion and activity of insulin cause diabetes mellitus (type I and II). The World Health Organization (WHO) reported that diabetes was the seventh most common disease worldwide, causing 1.5 million mortalities around the globe [7]. Insulin monomer contains two polypeptide chains, A and B, each consisting of 21 and 30 amino acids, respectively. These chains are held together by two interchains (A7–B7 and A20–B19) and one intrachain (A6–A11) disulfide bond. Additionally, insulin hexamer is stabilized by two Zn^2+^ ions whose diffusion rate is very poor in the bloodstream [8,9,10]. Here, under physiological conditions, the hexamer dissociated into insulin dimer. The insulin dimer breaks down into monomers that are biologically active and bind to the insulin receptor. Insulin dimer is stabilized by the presence of four hydrogen-bonding (H-bonding) interactions between Phe24 and Tyr26, which leads to the formation of anti-parallel β sheets.

(1) $$[\left(Ins{)}_{6}\;Zn_{2}.nH_{2}O\right]\;↔\;3{\left(Ins\right)}_{2}+2Zn+nH_{2}O\;↔\;6Ins+2Zn+nH_{2}O$$

Destructions of pancreatic β cells engendered the lower secretion of insulin, thereby resulting in type I diabetes, which is insulin dependent. Likewise, dimer formation plays a pivotal role in the storage and delivery of insulin [11,12]. Presently, insulin can be administrated through subcutaneous injection, but it fails in mimicking the endogenously secreted insulin. Moreover, it leads to various adverse effects such as the risk of hypoglycemia, hyperinsulinemia, atherosclerosis, and obesity [13]. An alternative route for both oral and transdermal insulin administration is achieved by exactly mimicking the secretion of endogenous insulin [14,15]. Meanwhile, the quest for effective oral insulin delivery began many years ago, but challenges such as enzymatic degradation within the gastrointestinal tract (GI), acidic environment in the stomach, drug absorption, solubility, molecular weight, and partition coefficients of physicochemical properties are involved. Additionally, oral insulin directly reaches the liver through the portal circulation, where immune tolerance of the GI is higher compared to the other routes of administration. Various drug-delivery strategies have been involved to improve the bioavailability of oral insulin [16,17].

Thus, ionic liquid (IL)-based drug formulations have received widespread attention to overcome the above-mentioned issues. ILs are composed of ions (cations and anions) that possess unique and tunable physicochemical properties. Recently, ILs have been widely utilized in the fields of biology and medicine (i.e., ioliomics) due to their biocompatibility [18]. Green solvent ILs can be employed in drug formulation and drug delivery, and also act as solvents/cosolvents for protein- and enzyme-based applications [19,20]. In addition to exhibiting more delightful properties such as the amelioration of solubility and absorption, ILs also function as carriers for protein and drugs. It has been found that the biocompatibility of choline cation confers biodegradability to all cholinium-based ILs [21,22,23]. Furthermore, cholinium aminoate ionic liquids stabilize the insulin structure, which was corroborated by MD simulations [24]. Choline and geranate (henceforth CAGE) ILs facilitate the transdermal drug delivery of BSA (bovine serum albumin), OVA (ovalbumin), and insulin by disrupting the stratum corneum (SC) of the skin. It is revealed in the literature that CAGE escalates the penetration ability of insulin around the skin, thereby causing a reduction in the blood glucose levels of rats. Thus, CAGE is exploited as a vehicle for transdermal drug delivery [25,26].

Hattori et al. demonstrated that the low bioavailability and water solubility of Nobiletin can be conquered by CAGE ILs, thus aiding transdermal administration [27]. ILs behave as a promising material for drug-delivery systems by potentially amplifying skin permeation with less irritation when compared to chemical permeation enhancers. Samir et al. studied the effective oral insulin formulations that have improved oral absorption and insulin stability and efficiently overcome the GI barrier. They evidenced, in in vivo and in vitro studies, high insulin-CAGE efficacy, biocompatibility, and long-term stability under room temperature [28]. CAGE is also used for the oral delivery of the water-insoluble drug sorafenib and to minimize the side effects [29,30]. Water molecules are surrounded the active sites of biomolecules such as protein, DNA, RNA, and membranes, referred to as biological water, which has a multifarious role in the packing and stabilization of protein structure [31,32]. In CAGE ILs, geranate anions reorganize to minimize the contacts of their hydrophobic tails with water up to a 0.65 water mole fraction, which is used for drug-delivery applications [33]. Recently, Palanisamy et al. studied the molecular mechanism of insulin-CAGE IL for the oral drug formulation, which reports that geranate anions have stronger interaction with the insulin surface than choline cation [34].

The consumption of ethanol modifies the glucose level and insulin metabolism in the human body. Still, the debate has promising attention paid towards the effect of the consumption of ethanol on diabetes mellitus, which is beneficial for modest consumption, with excess consumption leading to a risk of type II diabetes [35,36,37]. Ethanol facilitates diverse interactions, which also affects the cell processes and cell membrane. Insulin aggregates in the presence of ethanol, such as in a donut-shaped form. The inter-β-strand forms stronger H-bonding interactions, which leads to fibril formations [38]. Bagchi and his co-workers studied the insulin dimer dissociation in the presence of ethanol. They reported ethanol molecules to reduce the free energy barrier and increase the rate of dimer dissociation at 5% and 10% in water–ethanol mixtures. Ethanol is entered into the cavity of insulin hexamer and stabilized by forming an H-bond interaction with Glu-13. These molecules are in the cavity, which is directed by entropic stabilization, and the residence time is also very high. The importance of the molecular-level interactions of ethanol on insulin dimer was understood [39,40,41,42,43]. Mondal et al. reported that the two monomeric (center-of-mass) distances are not increased, but the number of contacts between two monomers sharply increased [44]. Karplus and his co-workers reported that the binding free energy of insulin dimerization is −11.2 kcal/mol, which is well correlated with the experimental value of −7.2 kcal/mol [45]. Changjun Chen et al. calculated the absolute binding free energy of insulin dimers to be −8.97 ± 1.41 kcal/mol, which is closer to the experimental value [46].

The main objective of this study is to unravel the interactions of insulin-CAGE formulations with and without ethanol molecules. This destabilizing agent influences the stability of insulin, whereas CAGE ILs strongly stabilize the insulin. The counteractions between CAGE ILs and ethanol on insulin were studied by using MD simulations. The stability of insulin was maintained up to 0.20 mole fraction of EtOH, after which the impact of ethanol on insulin was increased. Furthermore, we used two order parameters to study the insulin dimer dissociation by employing WT-MetaD simulations. We find the intermediate states of insulin dimer dissociation and the molecular interactions of ethanol with CAGE molecules. These observations concluded that ethanol molecules influence the stability of insulin, whereas CAGE ILs protect the structure of insulin dimer. Our study will provide insights into the stability of insulin dimer as well as the dissociation pathway during the incorporation of EtOH molecules.

## 2. Computational Details and Methods

### 2.1. Molecular Dynamics (MD) Simulations

**Figure 1:**
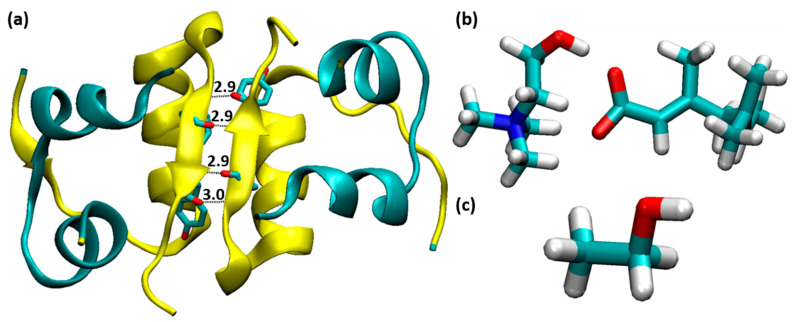
(**a**) The structure of insulin dimer, where chains A, C and B, D are indicated by cyan and yellow color, respectively (four H-bonds between Phe^B24^ and Tyr^B26^). (**b**) choline geranate IL and (**c**) ethanol molecule.

All the molecular dynamics (MD) simulations were performed using Gromacs 2020.4. The initial structure of the insulin dimer was retrieved from the protein data bank (PDB ID: 4INS) [47] at a resolution of 1.50 Å. The crystal structure contains the coordinates of the insulin dimer with two Zn atoms and water molecules. The Zn atoms and water molecules were removed, where Zn atoms were appropriate for insulin hexamer formation. We removed the B conformations of the residues Gly^B4^, Val^B12^, Glu^B21^, Arg^B22^, Thr^B27^, Arg^D22^, and Lys^D29^, where these residues contain two conformations (A and B) in the crystal structure of insulin. The resolved insulin dimer is solvated with a cubic box with SPC/E water models [48]. Different mole fractions of CAGE and ethanol molecules were added to the system, which is tabulated in Table 1. Furthermore, these systems were neutralized by Na^+^ ions. The force field parameters of Amber99SB-ILDN [49] were used for insulin and the general amber force field (GAFF) [50] was used for CAGE and ethanol. The structure of insulin dimer, CAGE, and ethanol were displayed in Figure 1. For all the systems, periodic boundary conditions (PBC) were applied for all three dimensions. Energy minimization was performed using the steepest descent algorithm. Furthermore, the system was equilibrated (5 ns) with NVT and NPT ensembles by employing a V-rescale thermostat and Berendsen barostat, respectively. All the systems were maintained at a constant temperature (T = 300 K) and pressure (1 bar). Thus, these equilibrated complexes were directly used as starting complexes for the MD study. The long-range electrostatic interactions were calculated using the Particle Mesh Ewald (PME) method [51,52], and the cut-off value of 1.2 nm was used for nonbonded van der Waals (vdWs) interactions. The leapfrog integrator [53] was used to integrate the equation of motion with a time step of 2 fs. All the hydrogen atoms were constrained using the LINCS algorithm [54]. Finally, the MD simulations were performed for 0.5 μs. We have extracted 2000 frames for further analysis, and these trajectories were visualized using pyMOL and VMD programs [55,56]. MMPBSA.py tool was used to calculate the binding free energy of insulin dimer [57].

### 2.2. Well-Tempered Metadynamics (WT-MetaD) Simulations

Metadynamics is the enhanced sampling method that is performed to unravel the protein dissociation and association [58,59]. In metadynamics simulations, the history-dependent bias potential, which is a function of collective variables (CVs), was used. We have employed well-tempered metadynamics (WT-MD) simulations to understand the insulin dimer dissociation by using the PLUMED 2.6 package [60]. Two order (CVs) parameters were used to calculate the dimer dissociation processes: (i) the distance between the center of mass (COM) of two monomers (R) and (ii) the number of contacts between C-alpha atoms of two monomers (Q) with a cut-off distance of 0.7 nm. A bias factor of 25.0 was used with the Gaussian hill height being 0.3 kJ/mol and hill widths being 0.03 and 1.0 nm. The simulations were performed at room temperature (300 K). We performed the biased simulations for 100 ns, and the convergence was confirmed by plotting the free energy profile with simulation time.

## 3. Results and Discussion

### 3.1. Structural Stability of Insulin Dimer with CAGE and Ethanol

**Figure 2:**
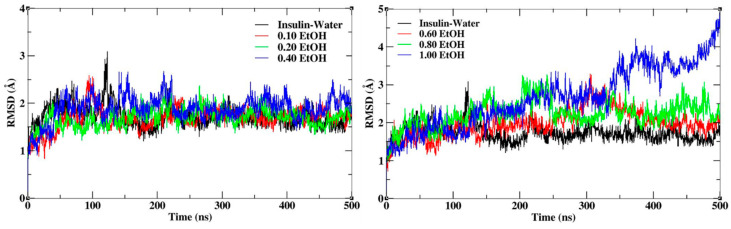
RMSD plots of insulin backbone with different mole fractions of EtOH.

Insulin dimer was stabilized during the MD simulation for all the systems with different mole fractions of EtOH. The root mean square deviation (RMSD) and fluctuations (RMSF) were calculated for insulin backbone atoms. RMSD calculates the conformational stability of insulin throughout the MD simulations. The RMSD plots of insulin with different mole fractions of EtOH are shown in Figure 2. The average RMSD value of insulin in the water medium is 1.7 Å, which was considered the reference value for all the systems. The average RMSD values were shown in Supplementary Materials Table S1. The higher conformational changes represent the structural destabilization, and the lower conformational changes represent the structural stabilization. The insulin structure was stabilized up to a 0.20 mole fraction of EtOH, while the gradual addition of ethanol influenced the conformational stability. In the figure displayed on the left side, slight deviations with respect to insulin dimer are observed at lower mole fractions of EtOH (0.10, 0.20 and 0.40) whereas the one on the right side with higher mole fractions of EtOH (0.60, 0.80 and 1.00) records larger deviations. This clearly shows that at a lower concentration of ethanol, the stability of the insulin structure is significantly maintained while the stability is reduced at higher concentrations of ethanol. Moreover, the largest deviations observed in 1.00 mole fraction of EtOH can be ascribed to the absence of the stabilizing agent CAGE ILs. We confirmed that CAGE ILs stabilized the insulin structure in the presence of ethanol molecules. RMSF calculates the conformational flexibility of each residue, where CAGE ILs and ethanol mixtures reduce the fluctuations. During the MD simulations, the C-terminal and N-terminal residues of chains A and B highly fluctuate. The mixtures of CAGE and ethanol molecules reduce the sharp fluctuations of the residues up to 0.20 mole fraction of EtOH, whereas the fluctuations were increased at higher mole fractions of EtOH. RMSF plots of insulin with different mole fractions of EtOH are shown in Supplementary Materials Figure S1. It confirms that the stability of insulin was maintained from 0.10 to 0.20 mole fractions of EtOH, after the addition of ethanol insulin stability was slightly reduced.

**Figure 3:**
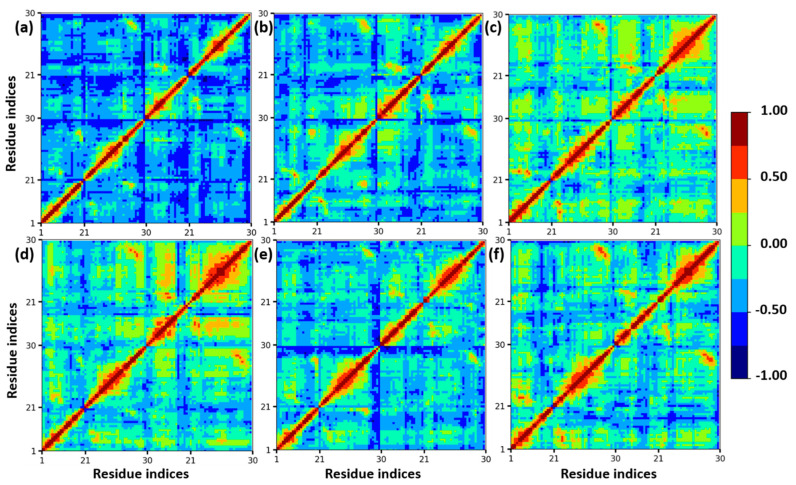
Calculated DCCM plots of insulin C−alpha atoms with different mole fractions of EtOH: (**a**) insulin–water, (**b**) 0.20, (**c**) 0.40, (**d**) 0.60, (**e**) 0.80, and (**f**) 1.00 mole fractions of EtOH. Color codes from red to blue represent the correlated and anti-correlated motions.

Furthermore, a dynamics cross-correlation map (DCCM) was calculated to understand the conformation changes of insulin dimer. In different mole fractions of EtOH, the proteins undergo conformational transition due to the motions of backbone atoms. DCCM was performed on the MD trajectories of insulin. DCCM plots show the collective motions of each residue, where highly correlated residues are in red, and the highly anti-correlated residues are in blue, which is displayed in Figure 3. These cross-correlation plots provide the dynamical information of C-alpha atoms of insulin dimer to all the systems. There are no significant changes in the β-sheets of chain B and chain D, whereas the loops and helices are slightly changed from anti-correlated motion to correlated motion (blue to red region). We confirmed that with the addition of ethanol, the structural stability of insulin was maintained due to the presence of CAGE ILs up to 0.20 mole fraction.

### 3.2. Accumulation of CAGE, Ethanol, and Water Molecules around the Insulin Dimer

Destabilizing agent ethanol affects the insulin stability by forming H-bonding and hydrophobic interactions due to its amphiphile nature. To gain more insight into the molecular interactions of CAGE and ethanol molecules with insulin, we calculated the accumulation of choline, geranate, and ethanol molecules on the insulin surface. We constrained the surface area within 3.4 Å of insulin as the first solvation shell to understand the interactions of choline, geranate, and ethanol. We calculated the accumulation propensities (***R_ion_***) of choline and geranate ILs, where the ***R_ion_*** is defined as the ratio between the average number of choline, geranate, and ethanol with water around the protein surface divided by the total number of choline, geranate, and ethanol with water in the simulation.

(2) $$R_{ion}=\frac{{\left(n_{ion}/n_{water}\right)}_{surface}}{{\left(n_{ion}/n_{water}\right)}_{total}}$$

With the addition of ethanol molecules to the system, the average number of geranate ions around the insulin was high to the extent of 0.40 mole fraction of EtOH. With the addition of EtOH, the accumulation of geranate ions on the insulin surface sharply decreased. After 0.20 mole fraction of EtOH, the accumulation of water molecules decreased and the ethanol molecules increased sharply. Interestingly, more geranate ions interacted on the insulin surface than the choline cation. Geranate ions formed H-bonding interactions with the positively charged amino acids and water, whereas choline made hydrophobic interactions on the insulin surface. The average numbers of choline, geranate, and ethanol molecules on the insulin surface are presented in Table 2. We reported that the stability of insulin strongly depends on the accumulation of geranate and water. The earlier findings also suggest that water-mediated H-bonding interaction with geranate anions plays an important role in the stability of insulin dimer [33]. The accumulation of ethanol, choline, and geranate on the insulin surface is shown in Supplementary Materials Figure S2. These snapshots were extracted from the cluster analysis, based on the clusters of insulin structure; we calculated the spatial arrangements of ethanol, choline, and geranate ions on the first solvation shell of insulin. We confirmed that geranate anions more strongly interacted with the insulin surface than the choline and ethanol molecules. Furthermore, the above RMS results show that the insulin stability was maintained to the extent of a 0.20 mole fraction of EtOH. Beyond that, ethanol slightly influences the stability of insulin, which was confirmed by the exclusion of choline, geranate, and water molecules from the proximity of insulin. From these results, we confirmed that the stability of insulin was reduced when the interactions of insulin–geranate ions were reduced.

The average number of H-bonding interactions of ethanol, choline, geranate, and water molecules with insulin was calculated, which is shown in Supplementary Materials Table S2. The interactions of choline and geranate ions were reduced during the addition of ethanol. Geranate anions formed more H-bonding with insulin due to the presence of the carboxylate (-COO^−^) group, while choline formed a fewer number of interactions. These results conclude that the interactions between choline and geranate were decreased when the addition of ethanol to insulin. At 0.50 mole fractions of EtOH, the average number of H-bonding between insulin–geranate and insulin–ethanol was ~8, which confirms that geranate anion and ethanol equally interacted with the insulin. This suggests that geranate anions reduce the interactions between ethanol and insulin by interacting with ethanol molecules.

### 3.3. Interactions of Choline, Geranate, and Ethanol with Water Molecules

**Figure 4:**
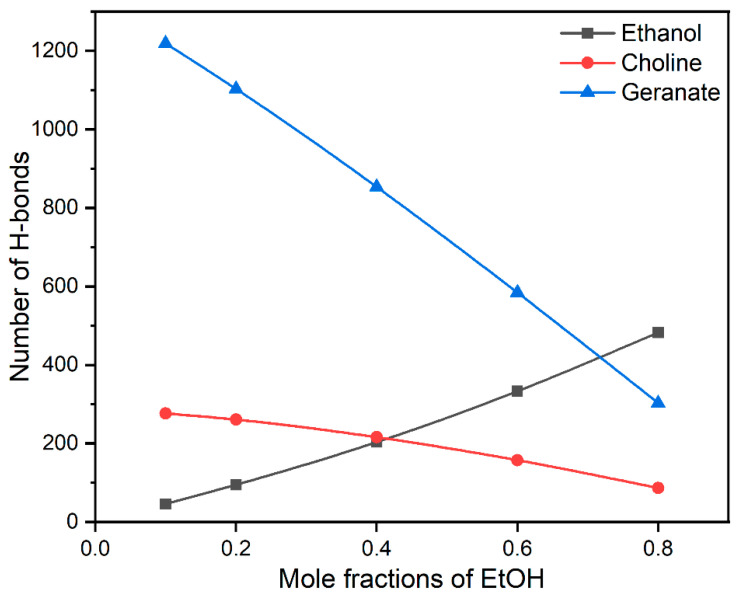
The average H-bonding interactions of ethanol, choline, and geranate with water molecules.

Water molecules play a pivotal role in the processes of protein association/dissociation. During the stabilization of insulin, water molecules are expelled from the first solvation shell of insulin. In the present study, ethanol molecules influence the stability of insulin by interacting with water molecules through H-bonds. In addition, water-mediated H-bonding interactions with CAGE ILs and hydrophilic part ethanol are helpful to the various structural formations. The earlier report suggests that the structure of CAGE ILs transitioned from lamellar phase to micellar phase at more than 67 vol % of water [61]. Similarly, we also found that micellar formations are more favorable for the stabilization of the insulin dimer. To interpret the role of water on the stability of insulin, we calculated the average number of H-bonding interactions of ethanol, choline, and geranate with water molecules, which are presented in Figure 4.

In general, CAGE ILs are found to establish a higher number of H-bonding interactions with water molecules than ethanol. So, the addition of ethanol facilitated a noticeable reduction in H-bonding interactions between CAGE ILs and water molecules, whereas a surge in the interactions between CAGE ILs and ethanol molecules was observed in Supplementary Materials Tables S3 and S4. After a 0.20 mole fraction of EtOH, the H-bonding between choline–water and geranate–water drastically reduced. Comparatively, geranate ions formed more H-bonding interactions than ethanol and choline due to the presence of the –COO^−^ group. We confirmed that geranate ions were stabilized through water-mediated H-bonding interactions, which are responsible for insulin stabilization. The choline cation (-OH group) causes fewer H-bonding interactions with water molecules due to the tertiary amino groups. In the case of ethanol, it causes a moderate number of H-bonding interactions and has hydrophobic (-CH_3_) and hydrophilic (-OH) groups. The average numbers of H-bonding interactions of choline, geranate, and ethanol with water molecules are shown in Supplementary Materials Table S3. We conclude that water-mediated H-bonding interactions stabilize the insulin structure. Surprisingly, geranate anions maintain their interactions with the insulin surface, when increasing the mole fractions of EtOH. This has arisen from the strong salt-bridge and hydrophobic interactions between geranate and insulin surface.

**Figure 5:**
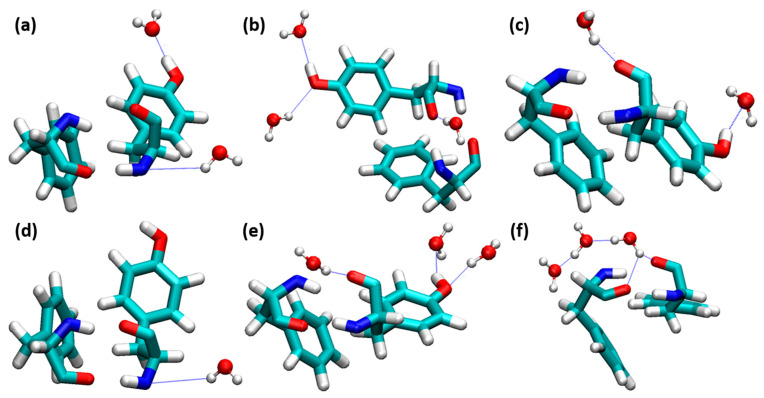
Water molecules interacted on the insulin dimer interface involving Phe^B24^-Tyr^D26^. (**a**) 0.10, (**b**) 0.20, (**c**) 0.40, (**d**) 0.60, (**e**) 0.80, and (**f**) 1.00 mole fractions of EtOH (Phe^B24^-Tyr^D26^ and water molecules are depicted in the licorice and ball-and-stick models, respectively. H-bonds are shown as blue dotted lines).

The interaction of water molecules are more responsible for the stabilization of insulin. Anti-parallel β-sheets contain Phe^24^, Phe^25^, and Tyr^26^, the presence of four H-bonding interactions between Phe^B24^ and Tyr^D26^ is responsible for the stabilization of insulin dimer. When water molecules interacted with the Phe^B24^-Tyr^D26^ residues, this directly influenced the stabilization of insulin dimer. We analyzed the interactions of water molecules between these residues, which are shown in Figure 5. Bridging-type water-mediated H-bonding interactions are responsible for the destabilization of insulin. In our study, the nonbridging H-bonding interactions exist in all the insulin dimers from 0.10 to 0.80 mole fractions of EtOH. Curiously, a water molecule interacted with the O atom of Phe^B24^ and Tyr^D26^ at a 1.00 mole fraction of EtOH, which leads to the structural deviations of insulin dimer. We concluded that nonbridging H-bonding interactions do not affect the stability of insulin dimer, as well as the dimerization. The changes in the H-bonding interactions between Phe^B24^ and Tyr^D26^ are displayed in Supplementary Materials Figure S3. Very strong H-bonding interactions were observed at 0.40 mole fraction of EtOH. The distances between four residues were increased after the addition of a 0.40 mole fraction of EtOH.

### 3.4. Interactions between CAGE and Ethanol

**Figure 6:**
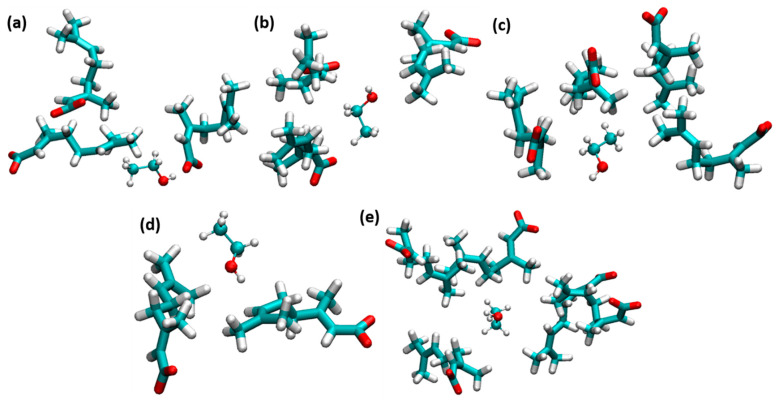
Ethanol molecules were trapped by geranate anions. (**a**) 0.10, (**b**) 0.20, (**c**) 0.40, (**d**) 0.60, and (**e**) 0.80 mole fractions of EtOH (licorice and ball-and-stick models indicate geranate and ethanol, respectively).

CAGE ILs strongly enhance the stability of insulin dimer through the water-mediated H-bonding interactions of geranate anions. The amphiphile group of ethanol strongly affects the hydrophobic regions of insulin. The counteraction between CAGE ILs and ethanol molecules was studied to unravel the stabilization of insulin dimer. With the addition of ethanol, H-bonding interactions of choline and geranate with ethanol molecules were increased to a 0.60 mole fraction of EtOH. Geranate anion is more responsible for the stabilization of insulin structure; it strongly interacted with ethanol. It contributes to electrostatic and vdWs interactions with the system. Ethanol molecules were trapped by the geranate anions, which is shown in Figure 6. Amphiphile group ethanol molecules form H-bonding and vdWs interactions with geranate anions.

Geranate anions form micellar-like structures and surround the ethanol molecules, which is more helpful for the stabilization of insulin dimer. The average number of H-bonding interactions of choline and geranate with ethanol is shown in Supplementary Materials Table S4. The amphiphilic group of choline geranate strongly interacted with the amphiphile group of ethanol. Interestingly, ethanol molecules are trapped between the micellar-like geranate anions. The insulin was stabilized by the reduction of interactions between ethanol and insulin. The radial distribution function (RDF) was calculated for choline (N1) and geranate (O2) with ethanol (O1) molecules. We observed a peak around r ~ 0.28 nm; a small peak represents the interactions between choline–ethanol, whereas a higher peak represents the interactions of geranate–ethanol. Our calculation suggests that ethanol molecules strongly interacted with the geranate anions than the counter ion. The interactions of choline–ethanol and geranate–ethanol exist around r ~ 0.3 nm, at the first solvation shell of protein. RDF plots of choline and geranate with water molecules are shown in Supplementary Materials Figure S4.

### 3.5. Binding Free Energy (∆G_binding_) Calculations

In this present work, the stability of insulin dimer with different mole fractions of EtOH was studied. The molecular mechanics generalized-born surface area (MM/GBSA) method was used to calculate the relative binding free energy of the insulin dimer by employing the MMPBSA.py tool. The experimentally calculated binding free energy of insulin dimer is −7.2 kcal/mol, which is correlated with the computationally calculated (enthalpic and entropic contributions) binding free energy is −11.9 kcal/mol. The relative binding free energy of insulin dimer with different mole fractions of EtOH was displayed in Table 3. The contributions of enthalpic energies are electrostatic, vdWs, generalized born, and surface area, and the energy is approximately −52.20 kcal/mol for insulin dimer in a pure water medium. It has more stable dimerization energy than the other systems of CAGE and ethanol mixtures. The total binding free energy predominantly depends on the energy of vdWs and solvation. The electrostatic energy changed with the addition of ethanol, which was similar for the two monomers. The individual contributions of binding free energies are presented in Supplementary Materials Table S5. In the presence of ethanol, there are no drastic changes in the enthalpy of all the systems. Dimerization enthalpic energies are similar for all the mole fractions of EtOH. In complete solvation of ethanol (1.00 mole fraction of EtOH), the electrostatic interactions dominantly improve the total binding free energy. At 0.60 mole fraction of EtOH is the least stable (~−47.62 kcal/mol).

Enthalpy plays a dominant role in the determination of the structure and stability of complexes during the solvent interaction process. At 0.40 mole fraction of EtOH, the accumulation of ethanol increased drastically, which resulted in the lower entropy value (Table 3). Furthermore, a significant decrease in entropy can be attributed to the lamellar and micellar phases formed between geranate and water at a 0.40 mole fraction of EtOH. Additionally, the enthalpic energy is decreased from 0.10 to 0.60 mole fractions of EtOH, after which the energy increased. Entropic contributions (−T (∆S_trans_ + ∆S_rot_ + ∆S_vib_)) were included for the total binding free energy of the insulin dimer. We found that there was a slight variability in the dimerization energy with different mole fractions of EtOH. Entropic contributions of translational and rotational energies are similar for all the insulin dimers, whereas the most important contributions of vibration energy were suddenly dropped at 0.60 mole fractions of EtOH. After adding the enthalpic and entropic energy, the range of total binding free energy ranged from −20 to −8 kcal/mol. The insulin dimer stability depended on the interactions of ethanol and geranate ions on the insulin structure. We reported that insulin dimer is stable in all the simulated systems of different mole fractions of EtOH. The dimerization free energy was −16.1 kcal/mol for a 0.20 mole fraction of EtOH, after which the free energy decreased.

### 3.6. Free Energy Landscape (FEL) of Insulin Dimer

**Figure 7:**
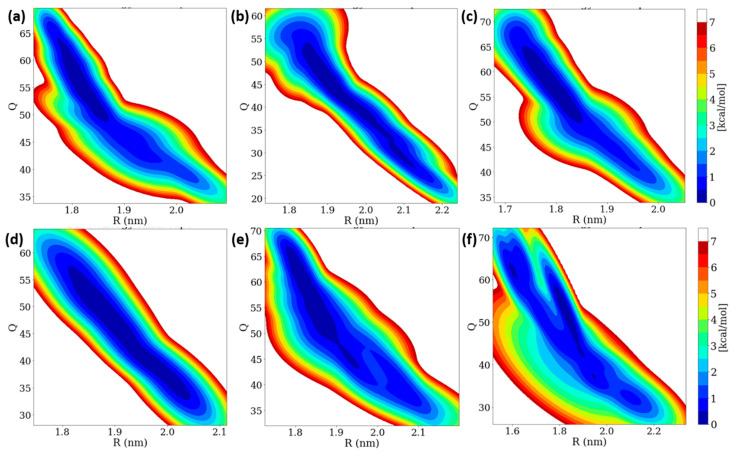
Two-dimensional free energy landscape of insulin dimer dissociation of (**a**) 0.10, (**b**) 0.20, (**c**) 0.40, (**d**) 0.60, (**e**) 0.80, and (**f**) 1.00 mole fractions of EtOH with two order parameters (R and Q).

Insulin dimer dissociation is the most important phenomenon, which is understood by using microscopic investigation. We performed a WT-MetaD simulation to study the rugged free energy landscape (FEL) of the insulin dimer by using two order parameters (CVs): (i) the distance between the center of mass (COM) of two monomers (R) and (ii) the number of contacts between the C-alpha atoms of two monomers (Q) with a cut-off distance of 0.7 nm. The FEL shows that the global minimum corresponds to the dimer (Q is ~50 and R is ~1.8 nm). Earlier studies also reported that the same initial R and Q values were observed [38,40]. In the dimer dissociation process, the number of contacts Q was sharply decreased, whereas the distances R were increased to 0.60 mole fractions of EtOH. After that, the number of contacts Q was increased while the distances R were decreased. The FEL contour of the insulin dimer is displayed in Figure 7. Interestingly, the insulin dimer was not dissociated, but distances between the two monomers were slightly increased. At a higher concentration of ethanol, the insulin dimer was dissociated into two monomers, whereas the concentration of ethanol was much less (300 molecules), so no dissociation occurred on the insulin dimer.

In WT-MetaD simulations, the dissociation process was characterized into three stages based on their energy basins in the R and Q parameters: (i) native state and (ii) two intermediate states (IS-1 and IS-2). The ranges of fluctuations of R and Q are displayed in Table S6. We observed that the α-helices moved away and β-sheet contacts were broken for 0.10 and 0.20 mole fractions of EtOH, which moved from NS to IS-2 region. Interestingly, ethanol molecules strongly interact with the four residues (Phe^24^ and Tyr^26^) during the trajectory at a 1.00 mole fraction of EtOH, whilst the H-bonds of four residues remain stable from 0.10 to 0.80 mole fractions of EtOH. We conclude that no dimer dissociation exists during the addition of ethanol, but the distances R between the two monomers were slightly increased.

## 4. Conclusions

The dissociation and association of insulin dimer are important biological processes. CAGE ILs strongly stabilized the insulin dimer, which can be exploited for oral and transdermal insulin delivery. From the present study, it has been evidenced that geranate ions form water-mediated H-bonding interactions on the insulin surface, which enhances its stability. The biased and unbiased MD simulations revealed that EtOH affected the stability of insulin dimer. Here, the stability of the dimer was found to be retained until a 0.20 mole fraction of EtOH, after which a decrease in structural stability was observed. Additionally, with a 1.00 mole fraction of EtOH (absence of CAGE ILs), a drastic decline was noticed. Moreover, the geranate anions exhibited stronger interactions with insulin than choline cation and EtOH molecules. The large number of H-bonding interactions of geranate with water molecules has been minimized with the inclusion of EtOH. Comparatively, EtOH established more H-bonding interactions with the geranate anion than the choline cation. Thus, geranate anions trap the EtOH molecules, thereby preventing the destabilizing interactions of EtOH on the insulin surface. Furthermore, calculation of dimerization free energy between the two monomers at 0.40 mole fraction of EtOH yielded the highest energy, which was reduced after excessive EtOH addition. FEL showed the absence of insulin dimer dissociation, whereas variations were noticed in both the number of contacts R and the distances Q. Subsequently, the current study concludes that geranate ions are more responsible for the stabilization of insulin dimer from the destabilizing EtOH molecules. Our results revealed that the administration of CAGE-insulin formulations was stable at a low concentration of EtOH. Thus, this study sheds light on the oral insulin delivery applications.

## Figures and Tables

**Table 1:** The system details of mole fractions of CAGE and ethanol molecules.

System	Mole Fraction of EtOH	EtOH	Choline	Geranate	Water
1	Ins-Water	0	0	0	8229
2	0.10	30	270	270	3748
3	0.20	60	240	240	4111
4	0.40	120	180	180	4791
5	0.60	180	120	120	5595
6	0.80	240	60	60	6330
7	1.00	300	0	0	7183

**Table 2:** The accumulation of choline, geranate, ethanol, and water molecules around the first solvation shell of the insulin dimer.

System	Mole Fraction of EtOH	EtOH	Choline	Geranate	Water	R_EtOH_	R_Choline_	R_Geranate_
1	0.10	2.79	27.60	36.27	256.15	1.35	1.49	1.96
2	0.20	6.93	25.09	31.56	279.39	1.71	1.54	1.93
3	0.40	16.83	21.96	33.34	274.84	2.44	2.13	3.23
4	0.60	20.20	11.87	19.64	218.12	2.88	2.54	4.20
5	0.80	28.13	6.86	13.84	237.49	3.12	3.06	6.19
6	1.00	38.66	00	00	297.66	3.11	---	---

**Table 3:** The binding free energy of insulin dimerization with different mole fractions of EtOH. Enthalpy, entropy, and binding free energies are indicated in kcal/mol.

Mole Fractions of EtOH	Enthalpy (∆H)	Entropy (∆S)	Total Binding Free Energy (∆G_bind_)
0.10	−49.69	−36.94	−12.75
0.20	−50.98	−34.88	−16.1
0.40	−49.32	−41.08	−8.24
0.60	−47.62	−33.88	−13.74
0.80	−50.22	−29.72	−20.5
1.00	−50.51	−38.50	−12.01
Ins-Water	−52.20	−34.70	−17.5

## Data Availability

Not applicable.

## Supplementary Materials

The following supporting information can be downloaded at: https://www.mdpi.com/article/10.3390/molecules27155031/s1, Figure S1: The RMSF plots of Insulin-water and different mole fractions of EtOH, Figure S2: The accumulation of (a) ethanol (b) choline and (c) geranate molecules on the first solvation shell of insulin. (0.10, 0.40 and 0.80 mole fractions of EtOH), Figure S3: The H-bonding interactions between Phe B24 and Tyr B26 along with distances (Å). (a) 0.10 (b) 0.20 (c) 0.40 (d) 0.60 (e) 0.80 (f) 1.00 mole fraction of EtOH, Figure S4: RDF plots of interactions of choline (N1), and geranate (O2) with ethanol (O1) molecules. (a) 0.10 (b) 0.20 (c) 0.40 (d) 0.60 (e) 0.80 mole fractions of EtOH. (Black and red indicates the choline and geranate, respectively). Table S1: The average RMSD, RMSF and Rg values of Insulin backbone with different mole fractions of EtOH, Table S2: The average number of H-bonding interactions of ethanol, choline, geranate and water with insulin dimer, Table S3: The average number of H-bonding interactions of ethanol, choline, geranate with water molecules, Table S4: The average number of H-bonding interactions of choline, geranate, water with ethanol molecules, Table S5: The binding free energy (in kcal/mol) of insulin dimerization with different mole fractions of EtOH. Electrostatic, vdWs, solvation and gas phase energy contributions of simulation systems, Table S6: The range of fluctuations of insulin dimer dissociation process in the R and Q.
