# Supplementary material for: Counteractive Effects of Choline Geranate (CAGE) ILs and Ethanol on Insulin’s Stability—A Leap Forward towards Oral Insulin Formulation
Source: Molecules. 2022 Aug 8;27(15):5031. doi: 10.3390/molecules27155031 (PMC9370287; doi:10.3390/molecules27155031)
Supplement: Supplementary file 1 [file molecules-27-05031-s001.zip › molecules-1832213-supplementary.pdf]

Supplementary Materials

Counteractive Effects of Choline Geranate (CAGE) ILs and Ethanol on Insulin's Stability - A Leap Forward Towards Oral Insulin Formulation

Kandhan Palanisamy[1] and Muthuramalingam Prakash[1,*]

[1]*Department of Chemistry, Faculty of Engineering and Technology, SRM Institute of Science and Technology, Kattankulathur 603 203, Chengalpattu, Tamil Nadu, India.*

*Email: prakashspm@gmail.com & prakashm4@srmist.edu.in

[†]**Supplementary Materials available:** The analysis of RMS, RDF, H-bonding interactions, individual binding free energies, and accumulation of ions on insulin surface.

Table S1. The average RMSD, RMSF and Rg values of Insulin backbone with different mole fractions of EtOH.

Mole fractions of EtOH	Backbone RMSD (Å)	Backbone RMSF (Å)
Insulin-Water	1.73	1.01
0.10	1.68	1.02
0.20	1.69	0.97
0.40	1.89	1.20
0.60	1.97	1.21
0.80	2.24	1.16
1.00	2.66	1.59

Table S2. The average number of H-bonding interactions of ethanol, choline, geranate and water with insulin dimer.

Mole fractions of EtOH	Ethanol	Choline	Geranate	Water
0.10	0.97	8.09	13.61	174.89
0.20	2.66	7.33	12.37	181.28
0.40	7.41	6.79	11.52	171.56
0.60	10.46	5.17	10.64	176.27
0.80	12.75	3.25	5.38	182.25
1.00	16.82	---	---	211.42

Table S3. The average number of H-bonding interactions of ethanol, choline, geranate with water molecules.

Mole fractions of EtOH	Ethanol	Choline	Geranate
0.10	45.80	276.66	1218.92
0.20	94.78	260.79	1103.30
0.40	203.42	216.04	853.46
0.60	333.17	157.61	584.23
0.80	482.51	86.51	302.61

Table S4. The average number of H-bonding interactions of choline, geranate, water with ethanol molecules.

Mole fractions of EtOH	Choline	Geranate	Water
0.10	2.01	6.67	45.80
0.20	3.64	12.01	94.78
0.40	5.26	18.75	203.42
0.60	5.16	19.23	333.17
0.80	3.11	14.10	482.51

Table S5. The binding free energy (in kcal/mol) of insulin dimerization with different mole fractions of EtOH. Electrostatic, vdWs, solvation and gas phase energy contributions of simulation systems.

Energy contributions	Mole Fraction of EtOH						Ins-Water
	0.10	0.20	0.40	0.60	0.80	1.00	
ΔE_{ele}	-21.67	-33.14	-61.63	-43.32	-58.41	-79.70	-68.34
ΔE_{vdWs}	-70.04	-73.60	-67.78	-64.25	-70.89	-67.59	-75.83
ΔE_{GB}	50.39	64.62	88.29	67.61	87.44	104.90	100.97
ΔE_{Surf}	-8.38	-8.85	-8.19	-7.66	-8.36	-8.12	-8.99
Enthalpy (ΔH)	-49.7	-50.97	-49.31	-47.62	-50.22	-50.51	-52.19
$-T(\Delta S_{trans})$	14.82	14.82	14.82	14.82	14.82	14.82	14.82
$-T(\Delta S_{rot})$	13.81	13.82	13.84	13.81	13.83	13.81	13.79
$-T(\Delta S_{vib})$	8.30	6.23	12.41	5.25	1.06	9.87	6.08
Entropy (ΔS)	36.93	34.87	41.07	33.88	29.71	38.50	34.69

Table S6. The range of fluctuations of insulin dimer dissociation process in the R and Q.

Three states	R (nm)	Q
Native state (NS)	1.75-1.85	45.0-55.0
Intermediate state 1 (IS-1)	1.70-1.75	25.0-35.0
Intermediate state 2 (IS-2)	1.90-2.10	18.0-22.0

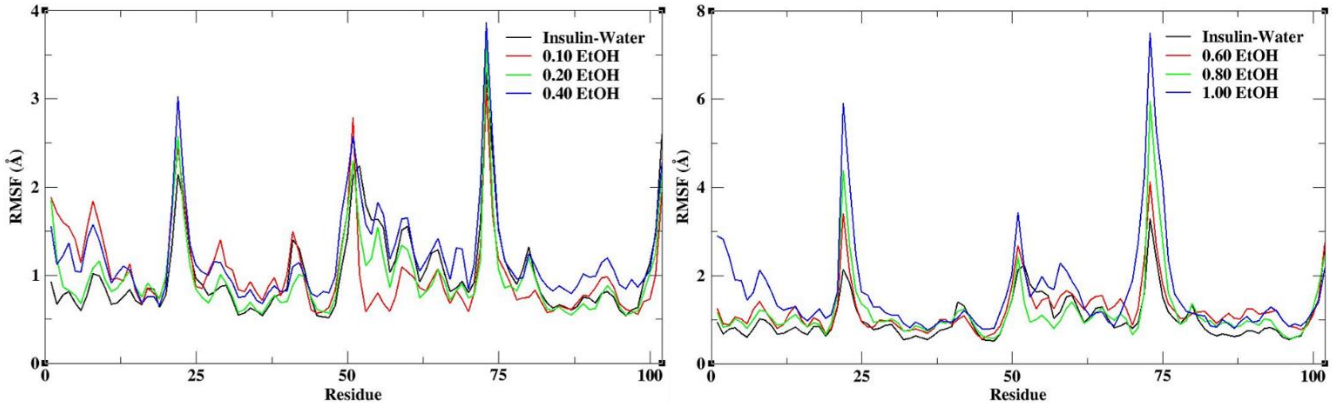

Figure S1. The RMSF plots of Insulin-water and different mole fractions of EtOH.

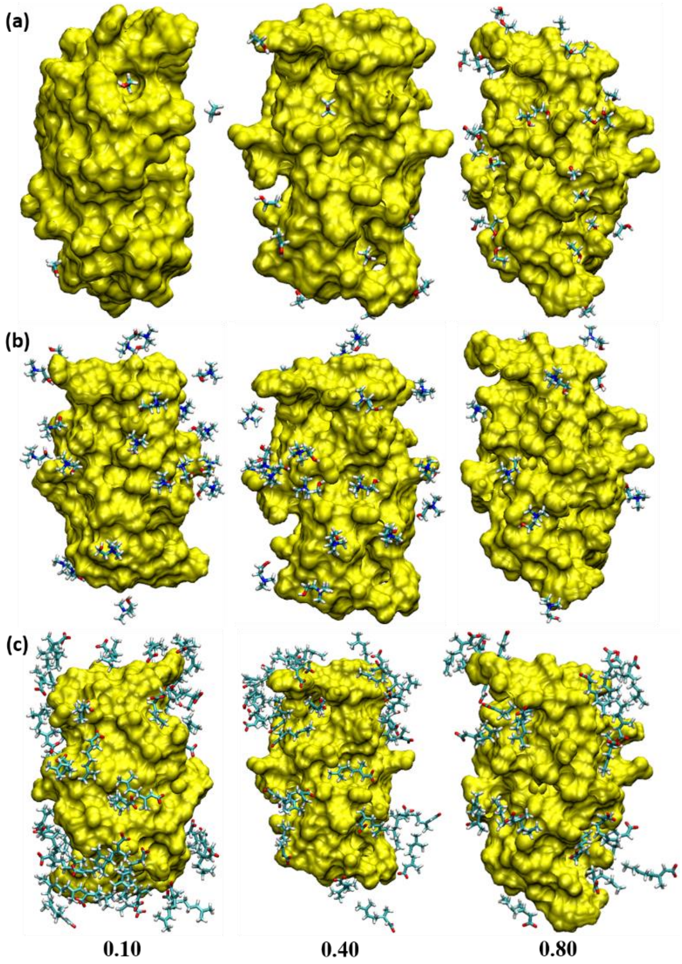

Figure S2. The accumulation of (a) ethanol (b) choline and (c) geranate molecules on the first solvation shell of insulin. (0.10, 0.40 and 0.80 mole fractions of EtOH)

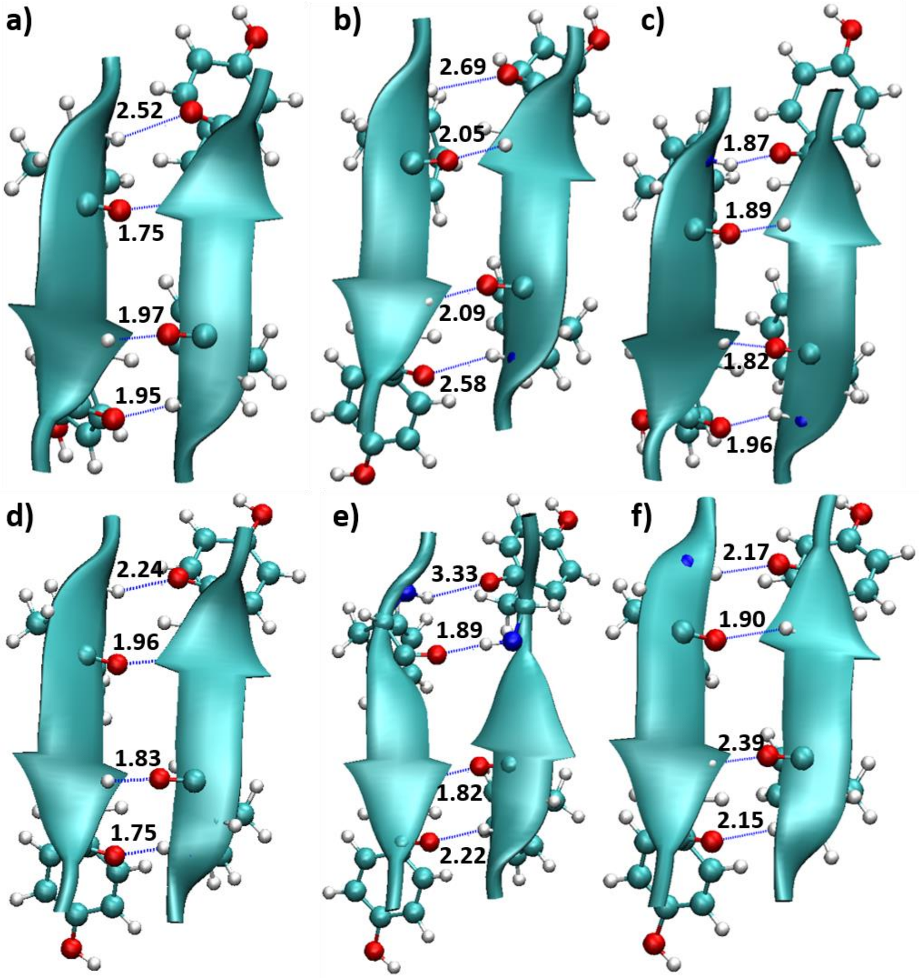

Figure S3. The H-bonding interactions between Phe^{B24} and Tyr^{B26} along with distances (Å). a) 0.10 b) 0.20 c) 0.40 d) 0.60 e) 0.80 f) 1.00 mole fraction of EtOH.

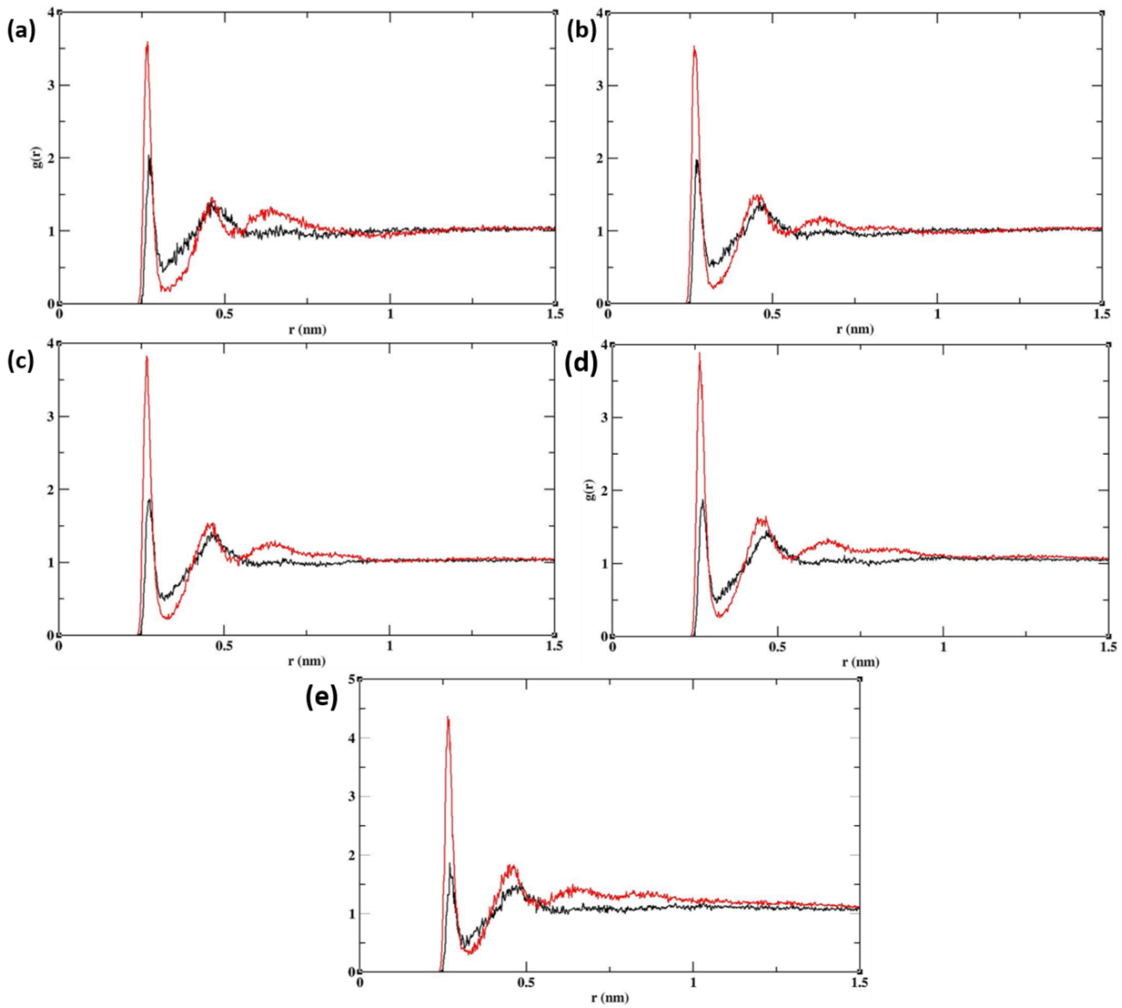

Figure S4. RDF plots of interactions of choline (N1), and geranate (O2) with ethanol (O1) molecules. (a) 0.10 (b) 0.20 (c) 0.40 (d) 0.60 (e) 0.80 mole fractions of EtOH. (Black and red indicates the choline and geranate, respectively)
